# Survival Outcome and Impact of Chemotherapy in T1 Node-Negative Triple-Negative Breast Cancer: A SEER Database Analysis

**DOI:** 10.1155/2020/8880727

**Published:** 2020-12-10

**Authors:** Jingyi Zhang, Wenna Wang, Jiayu Wang, Yang Luo, Shanshan Chen, Fei Ma, Binghe Xu, Ying Fan

**Affiliations:** Department of Medical Oncology, National Cancer Center/National Clinical Research Center for Cancer/Cancer Hospital, Chinese Academy of Medical Sciences and Peking Union Medical College, Beijing 100021, China

## Abstract

**Objective:**

Although triple-negative breast cancer (TNBC) has been considered to be an aggressive disease, the outcome of small-tumor (T1abcN0M0) TNBC and the effect of adjuvant chemotherapy on TNBC survival remain controversial.

**Methods:**

We identified 4565 T1abcN0M0 TNBC patients in the Surveillance, Epidemiology, and End Results (SEER) database from January 1, 2010, to December 31, 2015. After propensity score matching (PSM), 3214 patients were finally analyzed. Survival rates were compared among T1a, T1b, and T1c patients and between patients with and without adjuvant chemotherapy.

**Results:**

We classified 424, 1040, and 3101 cases as T1a, T1b, and T1c TNBC, respectively. A total of 2760 (60.5%) patients received adjuvant chemotherapy, accounting for 25.5%, 56.0%, and 66.8% of T1a, T1b, and T1c patients, respectively. Rates of 5-year breast cancer-specific survival (BCSS) for T1a, T1b, and T1c patients receiving chemotherapy were 97.8%, 94.1%, and 94.5%, respectively, compared with 97.2%, 94.0%, and 89.9% in patients without chemotherapy. Patients receiving adjuvant chemotherapy had higher 5-year BCSS (94.5% vs. 89.9%, *P* = 0.004) in the T1c subgroup, but no significant difference was detected in T1a or T1b patients due to adjuvant chemotherapy.

**Conclusion:**

Small-tumor TNBC showed very good prognosis. Adjuvant chemotherapy improved prognosis in T1c TNBC cases to a greater extent than in T1a and T1b patients. More large-scale clinical trials are needed, and further study should be conducted to determine appropriate adjuvant chemotherapy for T1c TNBC patients.

## 1. Introduction

Triple-negative breast cancer (TNBC) is defined immunohistochemically by the deficiencies of three receptors: estrogen receptor (ER), progesterone receptor (PR), and human epidermal growth factor receptor 2 (HER2). At present, TNBC comprises approximately 10–20% of all breast cancer patients [1]. This breast cancer subtype is a cause for great concern due to its poor prognosis [2], distinct metastatic patterns [3], and aggressive biological behavior [4, 5]. Because TNBC has no explicit molecular markers, chemotherapy is considered the backbone of TNBC treatment [6, 7]. Large-scale mammographic screening has increased the proportion of small-tumor detection from 36% to 68%; thus, breast cancer is increasingly diagnosed at the very early stage [8, 9]. Numerous studies have demonstrated that patients with T1a (1–5 mm), T1b (5–10 mm), and T1c (10–20 mm) node-negative tumors commonly have favorable prognosis [10]. Nevertheless, outcomes vary among different breast cancer subtypes, and those for very early-stage TNBC remain unclear. Therefore, although chemotherapy is recommended for TNBC, its benefit in the very early stage is not well delineated. In this study, we focused on survival outcomes in very early-stage TNBC based on information obtained from the large-scale Surveillance, Epidemiology, and End Results (SEER) database.

## 2. Materials and Methods

### 2.1. Data Source

All demographic and clinicopathological data were extracted from the SEER database, which is derived from 18 cancer registries across the United States (USA) and covers about 27.8% of incident cases in the USA [11]. The SEER database contains cancer-specific treatment profiles and survival data. Information used in the present study is based on the most recent follow-up data available (i.e., December 31, 2015).

### 2.2. Study Population

We exported a case list from the SEER database using the SEER Stat v.8.3.5 statistical software. Only primary breast cancer cases negative for ER, PR, and HER2 were eligible for inclusion in this study. The cohort was further limited to patients in stage T1abcN0M0 according to the 7th edition of the American Joint Committee on Cancer (AJCC) system. Patients were excluded if they had been diagnosed by a death certificate only or autopsy only or for an absence of treatment and survival data. A total of 4565 T1abcN0M0 TNBC cases from January 1, 2010, to December 31, 2015, qualified for inclusion in this study. This study was approved by independent ethics committees of Cancer Institute and Hospital, Chinese Academy of Medical Sciences. Since the present study is a database-based analysis rather than experimental research on humans, informed patient consent is not needed.

### 2.3. Propensity Score Matching (PSM)

PSM is a statistical method for avoiding selection bias in nonrandomized studies and can be applied to balance covariates between treatment and control groups [12]. To ensure well-balanced characteristics between the chemotherapy and no chemotherapy/unknown groups, we used the R v.3.6.1 software to evaluate propensity scores matched for age, race, marital status, year of diagnosis, laterality, primary site, histology, and grade. We performed 1 : 1 pairing according to similar propensity values, with a caliper value of 0.15. Following PSM, a total of 3214 patients were included in the propensity score-matched cohort.

### 2.4. Statistical Analyses

The patient distribution and clinicopathologic characteristics of chemotherapy and nonchemotherapy/unknown groups were evaluated using Pearson's *χ*2 test. Breast cancer-specific survival (BCSS) and overall survival (OS) were the outcomes of interest. BCSS was defined as the interval from the date of diagnosis to the date of breast cancer death, and OS was identified as that from diagnosis to death due to any cause. Kaplan–Meier methods were used to estimate OS and BCSS distribution, and log-rank tests were applied to compare survival distributions. We used *Z*-tests to compare 5-year OS and BCSS rates for T1a, T1b, and T1c tumors across both groups. We used univariate and multivariate Cox regression models to identify prognostic factors associated with OS and BCSS. Hazard ratios (HRs) and 95% confidence intervals (CIs) were calculated using Cox proportional hazard regression models. The effect of chemotherapy on OS and BCSS was determined by subgroup analysis. The HR, 95%CI, and *P* value of each subset were displayed as forest plots. We used the SPSS v.24.0 software (IBM SPSS Statistics for Windows) and R v.3.6.1 software (R Project for Statistical Computing) for all statistical analyses. All tests were two sided, and statistical significance was assessed at a level of *P* < 0.05.

## 3. Results

### 3.1. Patient Characteristics

We identified 4565 T1abcN0M0 TNBC patients in the SEER database and classified 424, 1040, and 3101 cases as stage T1a, T1b, or T1c TNBC, respectively. A total of 2760 (60.5%) patients received adjuvant chemotherapy, accounting for 25.5%, 56.0%, and 66.8% of T1a, T1b, and T1c patients, respectively. Following PSM, patients were distributed in two groups, treated with and without adjuvant chemotherapy; both treatment groups contained the same number of cases (1607 vs. 1607). Among 3214 patients identified, 328 (10.2%) patients were in T1a, 758 (23.6%) patients were in T1b, and 2128 (66.2%) patients were in T1c. In the matched dataset, 58 (17.7%), 363 (47.9%), and 1186 (55.7%) patients in stages T1a, T1b, and T1c of TNBC, respectively, received adjuvant chemotherapy. Generally, baseline data were comparable between the two groups after PSM (*P* > 0.05). Demographic features and clinicopathologic characteristics are listed in Table 1.

### 3.2. Survival Outcomes

**Figure 1:**
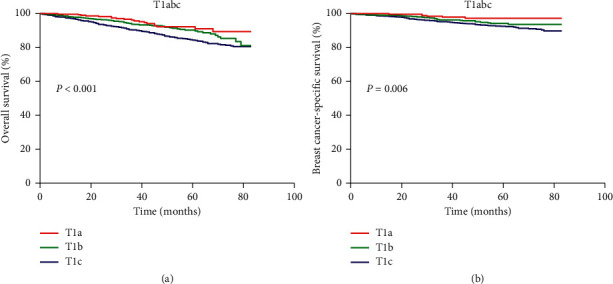
(a) Kaplan–Meier curves and log-rank test of overall survival in T1abcN0 triple-negative breast cancer. (b) Kaplan–Meier curves and log-rank test of breast cancer-specific survival in T1abcN0 triple-negative breast cancer.

**Figure 2:**
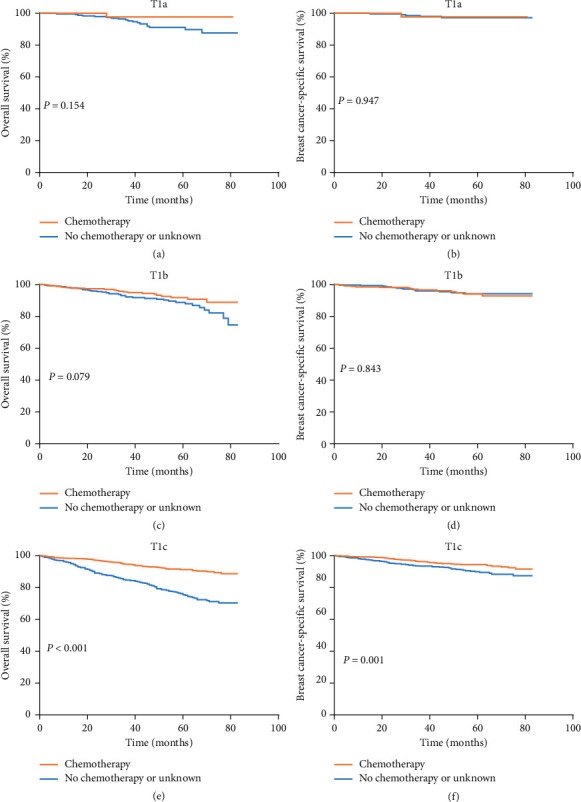
(a) Kaplan–Meier curves and log-rank test of overall survival in the T1a subgroup. (b) Kaplan–Meier curves and log-rank test of breast cancer-specific survival in T1a subgroup. (c) Kaplan–Meier curves and log-rank test of overall survival in T1b subgroup. (d) Kaplan–Meier curves and log-rank test of breast cancer-specific survival in the T1b subgroup. (e) Kaplan–Meier curves and log-rank test of overall survival in the T1c subgroup. (f) Kaplan–Meier curves and log-rank test of breast cancer-specific survival in the T1c subgroup.

The median follow-up time was 47 months. After PSM, 102 deaths were recorded in the chemotherapy group (*n* = 1607), among which 69 deaths were attributed to breast cancer. Among the chemotherapy-naïve/unknown group (*n* = 1607), 234 deaths were recorded, with 89 related to breast cancer. Survival curves according to chemotherapy treatment and tumor size are presented in Figures 1 and 2. The 5-year OS and BCSS rates of T1a patients were 92.9% and 97.3%, respectively; those of T1b TNBC patients were 90.2% and 94.1% and those of T1c patients were 84.3% and 92.6%. In T1a patients, chemotherapy and chemotherapy-naïve groups had significantly different 5-year OS rates (97.8% vs. 91.1%, *P* = 0.039), whereas no difference was detected in 5-year BCSS (97.8% vs. 97.2%, *P* = 0.388). In T1b patients, no significant difference in 5-year OS rates (91.9% vs. 88.6%, *P* = 0.195) or 5-year BCSS (94.1% vs. 94.0%, *P* = 0.399) was found between chemotherapy and chemotherapy-naïve groups. In the T1c subgroup, chemotherapy improved 5-year OS (91.4% vs. 75.5%, *P* < 0.001) and BCSS (94.5% vs. 89.9%, *P* = 0.004) (Table 2).

### 3.3. Univariate and Multivariate Analyses

The results of univariate and multivariate survival analyses are shown in Tables 3 and 4. On the basis of univariate Cox regression hazard analysis, substage T1c was associated with worse OS (HR = 2.105; 95%CI, 1.321–3.355; *P* = 0.002) and BCSS (HR = 3.160; 95%CI, 1.392–7.173; *P* = 0.006). Absence of adjuvant chemotherapy was associated with poor OS (HR = 2.445; 95%CI, 1.938–3.085; *P* < 0.001) and BCSS (HR = 1.374; 95%CI, 1.003–1.882; *P* = 0.048). In the multivariate model, substage T1c predicted worse OS (HR = 2.742; 95%CI, 1.702–4.418; *P* < 0.001) and BCSS (HR = 3.550; 95%CI, 1.542–8.173; *P* = 0.003) after adjusting for other prognostic factors. A lack of adjuvant chemotherapy predicted poor OS (HR = 2.766; 95%CI, 2.185–3.501; *P* < 0.001) and BCSS (HR = 1.615; 95%CI, 1.174–2.222; *P* = 0.003).

### 3.4. Subgroup Analysis

**Figure 3:**
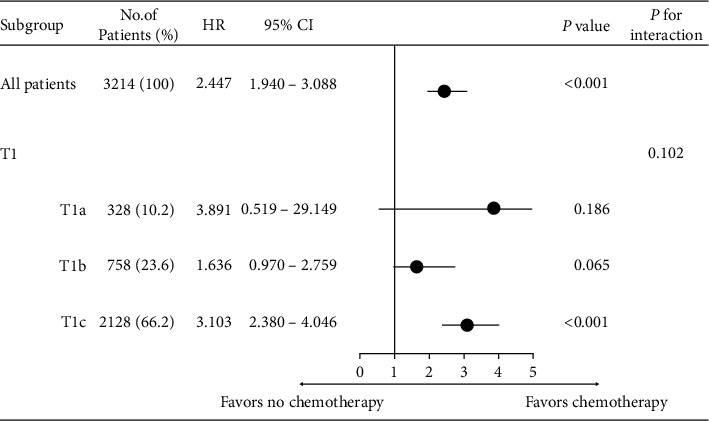
Forest plot of hazard ratios (HRs) and 95% confidence intervals (CIs) of overall survival among subgroups.

**Figure 4:**
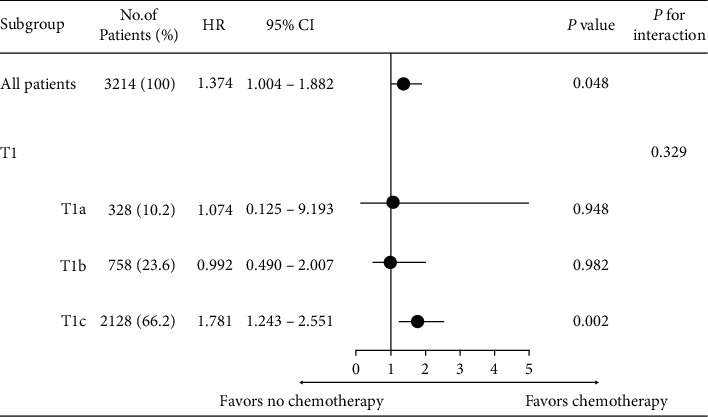
Forest plot of hazard ratios (HRs) and 95% confidence intervals (CIs) of breast cancer-specific survival among subgroups.

We performed exploratory subgroup analyses to explore further the effects of prognostic factors; the results are shown as forest plots of HRs and 95%CIs for OS (Figure 3) and BCSS (Figure 4). OS increased significantly when chemotherapy was performed in T1cN0 TNBC patients (HR = 3.103; 95%CI, 2.380–4.046; *P* < 0.001) and BCSS (HR = 1.781; 95%CI, 1.243–2.551; *P* = 0.002). However, T1a and T1b TNBC patients did not benefit from chemotherapy treatment in terms of either OS or BCSS.

## 4. Discussion

TNBC is considered to be an aggressive breast cancer subtype due to its worse prognosis, even in the early stages [13]. Systemic adjuvant chemotherapy is currently the only treatment for early stage TNBC. The application of adjuvant chemotherapy has been increasing even among T1 node-negative TNBC patients [14]. According to the data used in this study, chemotherapy treatment was applied to 60.5% of T1N0M0 TNBC patients, accounting for 25.5%, 56.0%, and 66.8% of stage T1a, T1b, and T1c patients, respectively. Therefore, a categorical increase in tumor size was associated with a progressive decrease in survival outcome for very early stage small node-negative disease.

Recommendations for adjuvant chemotherapy in patients with early stage TNBC were updated for the current NCCN guidelines (2018.V3), which advise no adjuvant therapy for stage T1aN0 TNBC, consideration of chemotherapy for the stage T1bN0 subgroup, and recommending chemotherapy treatment to stage T1cN0 cases [6]. The 2019 St.Gallen guidelines [15] recommend that patients with tumor size > 0.5 cm should be provided adjuvant chemotherapy treatment and that adjuvant chemotherapy for T1aN0 tumors should be decided on a case by case basis. It remains highly uncertain whether adjuvant chemotherapy benefits TNBC patients with small node-negative tumors.

Several studies have reported good outcomes in T1abN0 TNBC patients treated without chemotherapy [16, 17]. A large observational study included 4113 cases via the National Comprehensive Cancer Network, involving 363 stage T1ab TNBC patients, and reported that TNBC patients untreated with chemotherapy had 5-year distant relapse-free survival (DRFS) rates of 93% for T1a tumors and 90% for T1b tumors. The 5-year DRFS for patients treated with chemotherapy was 100% for T1a TNBC and 96% for T1b TNBC [13]. Some other studies have also reported that adjuvant chemotherapy does not improve T1abN0 TNBC prognosis [18–21]. In the present study, we concentrated on T1 node-negative TNBC and separated stage T1 tumors into substages T1a, T1b, and T1c. In our comparison of survival rates between chemotherapy and nonchemotherapy/unknown groups, significant differences were observed only in T1c cases. Consistent with our results, Ren et al. [22] suggested that chemotherapy is likely to be inappropriate for T1b patients, implying that it may be better to reduce chemotherapy for this substage. In this context, it is possible that T1cN0M0 TNBC patients need chemotherapy, while T1abN0M0 TNBC patients may not need. However, viewpoints on chemotherapy in T1N0 TNBC patients differ due to the reported high risk of recurrence in T1b tumors, with some researchers suggesting that T1bN0 TNBC patients should accept adjuvant systemic treatment [23–25]. Such studies may have been subject to underestimation in their results, given their small sample sizes and rather limited number of recurrences for very early-stage disease. There remains a need for further randomized prospective studies to resolve this controversy.

Although patients with T1ab TNBC may not benefit from chemotherapy, 108 T1a cases (25.5%) and 582 T1b cases (56.0%) within the cohort received adjuvant chemotherapy. Among these cases, 95.4% and 94.7% of patients treated with chemotherapy for T1a and T1b TNBC actually had poorly to moderately differentiated tumors. The 5-year OS rate for T1a tumors treated with chemotherapy was better than that of the chemotherapy-naïve group (97.8% vs. 91.1%, *P* = 0.039), which was inconsistent with our conclusion; however, of the 16 deaths recorded in the chemotherapy-naïve group, 11 deaths were attributed to causes other than breast cancer.

This retrospective study was conducted based on the large, well-established, standardized populations of the SEER database. As the first study to be conducted using PSM for small, node-negative TNBC, our results are more reliable than those of studies performed without the benefit of PSM. Limitations of this study included the absence of a molecular marker such as Ki-67, systemic chemotherapy regimens, and recurrence data. Especially, due to the lack of systemic therapy information, we did not compare efficacy of the intensive chemotherapy with other de-escalating chemotherapy in T1N0M0 TNBC patients. Therefore, further prospective randomized studies are needed to provide evidence that whether this group of patients should be treated with less-intensive chemotherapy. Additionally, the recurrence data of this very early-stage disease were not available, and consequently, our study was unable to demonstrate the role of chemotherapy in reducing the recurrence rate for T1N0M0 TNBC patients. Other limitations included the lack of information on rare subtypes of TNBC that could alter therapy such as metaplastic, adenoid cystic and apocrine subtype, the absence of central confirmation of TNBC status, and the absence of information on TILs. Moreover, the study cohort included few incidents and limited follow-up time, which may have led to biases.

## 5. Conclusions

We conducted a retrospective cohort study using cases extracted from the SEER database to determine the effect of chemotherapy in T1abcN0M0 TNBC patients. Our results indicate that T1cN0 TNBC patients have improved survival while receiving chemotherapy, however, TNBC patients with T1a and T1b tumors may not obtain similar benefits from chemotherapy. Further clinical trials are needed to verify these findings.

## Figures and Tables

**Table 1:** Patient characteristics of the study population.

	Original dataset			Matched dataset		
	Chemotherapy (*n* = 2760)(%)	No chemotherapy or unknown (*n* = 1805)(%)	*P*	Chemotherapy (*n* = 1607)(%)	No chemotherapy or unknown (*n* = 1607)(%)	*P*
Age (years)			<0.001			1.000
≤50	1022 (37.0)	271 (15.0)		268 (16.7)	269 (16.7)	
>50	1738 (63.0)	1534 (85.0)		1339 (83.3)	1338 (83.3)	
Race			0.812	0.725		
White	2067 (74.9)	1351 (74.8)		1181 (73.5)	1174 (73.1)	
Black	454 (16.4)	289 (16.0)		281 (17.5)	275 (17.1)	
Other/unknown	239 (8.7)	165 (9.1)		145 (9.0)	158 (9.8)	
Year of diagnosis			<0.001			0.872
2010-2011	826 (29.9)	663 (36.7)		565 (35.2)	555 (34.5)	
2012-2013	980 (35.5)	621 (34.4)		568 (35.3)	565 (35.2)	
2014-2015	954 (34.6)	521 (28.9)		474 (29.5)	487 (30.3)	
Laterality			0.963			0.480
Left	1413 (51.2)	922 (51.1)		839 (52.2)	818 (50.9)	
Right	1347 (48.8)	883 (48.9)		768 (47.8)	789 (49.1)	
Primary site			<0.001			0.951
Nipple/central	73 (2.6)	82 (4.5)		53 (3.3)	62 (3.9)	
UIQ	472 (17.1)	261 (14.5)		236 (14.7)	247 (15.4)	
LIQ	192 (7.0)	112 (6.2)		112 (7.0)	109 (6.8)	
UOQ	982 (35.6)	588 (32.6)		541 (33.7)	534 (33.2)	
LOQ	214 (7.8)	135 (7.5)		128 (8.0)	124 (7.7)	
Other	827 (30.0)	627 (34.7)		537 (33.4)	531 (33.0)	
Histologic subtype			<0.001			0.144
Ductal	2464 (89.3)	1488 (82.4)		1381 (85.9)	1341 (83.4)	
Lobular	16 (0.6)	21 (1.2)		16 (1.0)	20 (1.2)	
Other	280 (10.1)	296 (16.4)		210 (13.1)	246 (15.3)	
Grade			<0.001			0.101
1-2	548 (19.9)	671 (37.2)		444 (27.7)	503 (31.3)	
3	2156 (78.1)	1078 (59.7)		1119 (69.6)	1054 (65.6)	
Unknown	56 (2.0)	56 (3.1)		44 (2.7)	50 (3.1)	

Abbreviations indicate the breast quadrant. UIQ, upper-inner; LIQ, lower-inner; UOQ, upper-outer; LOQ, lower-outer.

**Table 2:** T1 tumor survival outcomes of patients following chemotherapy treatment.

Outcome	Chemotherapy			No chemotherapy or unknown			*P*
	5-year estimate (%)	95% CI	Total no. of events	5-year estimate (%)	95% CI	Total no. of events	
T1a							
OS	97.8	93.5–100.0	1	91.1	86.8–95.4	16	0.039
BCSS	97.8	93.5–100.0	1	97.2	94.7–99.7	5	0.388
T1b							
OS	91.9	88.2–95.6	20	88.6	84.7–92.5	32	0.195
BCSS	94.1	90.8–97.4	14	94.0	91.1–96.9	16	0.399
T1c							
OS	91.4	89.4–93.4	73	75.5	72.0–79.0	166	<0.001
BCSS	94.5	92.9–96.1	47	89.9	87.4–92.4	64	0.004

Abbreviations: OS, overall survival; BCSS, breast cancer-specific survival; CI, confidence interval.

**Table 3:** Univariate and multivariate analyses of overall survival.

Variables	Univariate analysis			Multivariate analysis		
	HR	95% CI	*P*	HR	95% CI	*P*
Age (years)						
>50 vs. ≤ 50	2.272	1.543–3.345	<0.001	2.227	1.507–3.289	<0.001
Histologic subtype						
Lobular vs. ductal	0.905	0.337–2.429	0.843	0.864	0.317–2.356	0.775
Tumor size						
T1a	1			1		
T1b	1.415	0.846–2.369	0.186	1.680	0.998–2.829	0.051
T1c	2.105	1.321–3.355	0.002	2.742	1.702–4.418	<0.001
Grade						
3 vs. 1–2	1.213	0.946–1.555	0.128	1.153	0.891–1.491	0.278
Chemotherapy						
No/unknown vs. yes	2.445	1.938–3.085	<0.001	2.766	2.185–3.501	<0.001

Abbreviations: HR, hazard ratio; CI, confidence interval.

**Table 4:** Univariate and multivariate analyses of breast cancer-specific survival.

Variables	Univariate analysis			Multivariate analysis		
	HR	95% CI	*P*	HR	95% CI	*P*
Age (years)						
>50 vs. ≤ 50	1.599	0.979–2.613	0.061	1.686	1.027–2.769	0.039
Histologic subtype						
Lobular vs. ductal	0.000	0.000	0.948	0.000	0.000	0.948
Tumor size						
T1a	1			1		
T1b	2.281	0.951–5.466	0.065	2.462	1.019–5.950	0.045
T1c	3.160	1.392–7.173	0.006	3.550	1.542–8.173	0.003
Grade						
3 vs. 1–2	1.461	0.997–2.140	0.052	1.246	0.845–1.838	0.268
Chemotherapy						
No/unknown vs. yes	1.374	1.003–1.882	0.048	1.615	1.174–2.222	0.003

Abbreviations: HR, hazard ratio; CI, confidence interval.

## Data Availability

All data generated or analyzed during this study are included in the following site: https://seer.cancer.gov/. Requests for material should be made to the corresponding author.
